# Identification and structure elucidation of the pro‐resolving mediators provides novel leads for resolution pharmacology

**DOI:** 10.1111/bph.14336

**Published:** 2018-06-03

**Authors:** Jesmond Dalli, Charles N Serhan

**Affiliations:** ^1^ Lipid Mediator Unit, William Harvey Research Institute, Barts and the London School of Medicine Queen Mary University of London London UK; ^2^ Center for Experimental Therapeutics and Reperfusion Injury, Department of Anaesthesia, Perioperative and Pain Medicine, Building for Transformative Medicine Brigham and Women's Hospital and Harvard Medical School Boston MA USA

## Abstract

Inflammatory diseases are a major socio‐economic burden, with the incidence of such conditions on the rise, especially in western societies. For decades, the primary treatment paradigm for many of these conditions was to develop drugs that inhibit or antagonize the production and biological actions of molecules that were thought to be the culprits in propagating disease; these include cytokines and eicosanoids. This approach is effective in controlling disease propagation; however, long‐term exposure to these anti‐inflammatories is also associated with many side effects, some of which are severe, including immune‐suppression. The discovery that termination of self‐limited acute inflammation is an active process orchestrated by endogenous mediators, including the essential fatty acid‐derived resolvins, protectins and maresins, has provided novel opportunities for the design of therapeutics that control inflammation with a lower burden of side effects. This is because at variance to anti‐inflammatories, pro‐resolving mediators do not completely inhibit inflammatory responses; instead, these mediators reprogramme the immune response to accelerate the termination of inflammation, facilitating the regain of function. The scope of this review is to highlight the biological actions of these autacoids and their potential utility as lead compounds in developing resolution pharmacology‐based therapeutics.

**Linked Articles:**

This article is part of a themed section on Eicosanoids 35 years from the 1982 Nobel: where are we now? To view the other articles in this section visit http://onlinelibrary.wiley.com/doi/10.1111/bph.v176.8/issuetoc

**Abbreviations**
13‐HDPA: 13‐(R)‐hydroxy‐7Z,10Z,13R,14E,16Z,19Z‐docosapentaenoic acid
13‐HpDPA: 13‐(R)‐hydroxyperoxy‐7Z,10Z,13R,14E,16Z,19Z‐docosapentaenoic acid
13S, 14S‐epoxy‐MaR: 13S,14S‐epoxy‐4Z,7Z,9E,11E,16Z,19Z‐docosahexaenoic acid
17R‐RvD3: 4S,11R,17R‐trihydroxy‐5Z,7E,9E,13Z,15E,19Z‐docosahexaenoic acid
ALX: lipoxin A_4_ receptor
BLT1 receptor: LTB_4_ receptor
DHA: docosahexaenoic acid
DSS: dextran sodium sulphate
EPA: eicosapentaenoic acid
LOX: lipoxygenase
LXA_4_: 5S,6R,15S‐trihydroxy‐7E,9E,11Z,13E‐eicosatetraenoic acid
MaR: maresin
MaR1: 7R,14S‐dihydroxy‐4Z,8E,10E,12Z,16Z,19Z‐docosahexaenoic acid
MaR1_n‐3 DPA_: 7R,14Sdihydroxy‐8E,10E,12Z,16Z,19Z‐docosapentaenoic acid
MaR2: 13R,14S‐dihydroxy‐4Z,7Z,9,11,16Z,19Z‐docosahexaenoic acid
MaR_n‐3 DPA_: n‐3docosapentaenoic acid‐derived maresins
MCTR: maresin conjugates in tissue regeneration
MCTR1: 13R‐glutathionyl,14S‐hydroxy‐4Z,7Z,9E,11E,13R,14S,16Z,19Z‐docosahexaenoic acid
MCTR2: 13R‐cysteinylglycinyl,14S‐hydroxy‐4Z,7Z,9E,11E,13R,14S,16Z,19Z‐docosahexaenoic acid
MCTR3: 13R‐cysteinyl,14S‐hydroxy‐4Z,7Z,9E,11E,13R,14S,16Z,19Z‐docosahexaenoic acid
miRNA: microRNA
n‐3 DPA: n‐3 docosapentaenoic acid
NALP3: NACHT, LRR and PYD domains‐containing protein 3
NSAIDs: non‐steroidal anti‐inflammatory drugs
PCTR: protectin conjugates in tissue regeneration
PCTR1: 16R‐glutathionyl,17S‐hydroxy‐4Z,7Z,10Z,12E,14E,19Z‐docosahexaenoic acid
PD: protectin
PD1: 10R,17S‐dihydroxy‐4Z,7Z,11E,13E,15Z,19Z‐docosahexaenoic acid
PD1_n‐3 DPA_: 10R,17S‐dihydroxy‐7Z,11E,13E,15Z,19Z‐docosapentaenoic acid
PD_n‐3 DPA_: n‐3 docosapentaenoic acid‐derived protectin
RCTR: resolvin conjugates in tissue regeneration
Rv: resolvin
RvD: docosahexaenoic acid‐derived resolvins
RvD1: 7S,8R,17S‐trihydroxy‐4Z,9E,11E,13Z,15E,19Z‐docosahexaenoic acid
RvD2: 7S,16R,17S‐trihydroxy‐4Z,8E,10Z,12E,14E,19Z‐docosahexaenoic acid
RvD3: 4S,11R,17S‐trihydroxy‐5Z,7E,9E,13Z,15E,19Z‐docosahexaenoic acid
RvD4: 4S,5R,17S‐trihydroxy‐6E,8E,10Z,13Z,15E,19Z‐docosahexaenoic acid
RvD5: 7S,17S‐dihydroxy‐4Z,8E,10Z,13Z,15E,19Z‐docosahexaenoic acid
RvD_n‐3 DPA_: n‐3 docosapentaenoic acid‐derived resolvins
RvE: eicosapentaenoic acid derived resolvins
RvE1: 5S,12R,18R‐trihydroxy‐6Z,8E,10E,14Z,16E‐eicosapentaenoic acid
RvE2: 5S,18R‐trihydroxy‐6E,8Z,11Z,14Z,16E‐eicosapentaenoic acid
RvE3: 17,18‐dihydroxy‐5Z,8Z,11Z,13E,15E‐eicosapentaenoic acid
RvT: thirteen series resolvins
SPM: specialized pro‐resolvin mediators
TRPV1: transient receptor potential cation channel subfamily V member 1

During evolution, multicellular organisms arose and cells within an organism became specialized to perform specific tasks leading to the formation of organs and tissues. These specialized organs required protection from invading microbes as well as mechanisms to repair and regenerate damaged tissues and organs, thus paving the way to the evolution of the immune system (Malagoli, 2016). In higher organisms, the immune system is divided into two arms, the innate arm provides the front line defence system and the adaptive arm provides protection from insults and/or pathogens to which the body was previously exposed. In some instances, immune responses may become dysregulated leading to disease. It is now appreciated that a significant portion of diseases that afflict western societies are associated with unbridled inflammation leading to damage of vital organs and tissues resulting in malaise and ultimately death (Kumar *et al*., 2014; Majno, 1991).

Classically, the inflammatory response has been divided into two phases, the initiation and resolution (or termination) phases (Kumar *et al*., 2014). Many studies conducted primarily in the last century focused on determining the mechanisms and molecules produced during the initiation phase, demonstrating that this was a tightly orchestrated response with the production of several classes of molecules that are involved in the recruitment of different leukocyte subsets and include the cytokines, chemokines and the classic eicosanoids (Kumar *et al*., 2014). Interested readers on the biology of these mediators are directed to articles in this special edition as well as to Samuelsson (2012) and Dinarello and Joosten (2016). The identification of these molecules also paved the way to the development of innovative therapeutics for the treatment of many inflammatory diseases that revolutionized medicine and medical practice. The drugs were designed to inhibit or antagonize the biological actions and the production of local mediators that were identified to be important in disease onset and progression. Longitudinal studies on the use of many of these therapeutics demonstrate that while these molecules are effective at inhibiting inflammation, they also come with many side effects. For example, non‐steroidal anti‐inflammatory drugs (NSAIDs) lead to an increased incidence of gastrointestinal bleeds (Goldstein and Cryer, 2015), and anti‐TNF increase the incidence of infections (Minozzi *et al*., 2016). Thus, these observations underscore an urgent need for the development of alternative approaches, especially in the treatment of chronic diseases, which would not interfere with the immune system and would carry a lower burden of side effects.

For many years, termination of inflammation was thought to occur *via* the simple dilution of inflammatory signals from the site of injury or infection, leading to the egress of white blood cells and re‐establishment of tissue function (Robbins and Cotran, 1979). Studies focusing on mechanisms that control the termination of inflammation demonstrated that arachidonic acid is not only a substrate in the biosynthesis of inflammation‐initiating molecules, such as PGs and leukotrienes, but is converted to protective and anti‐inflammatory molecules that include the lipoxins (Serhan *et al*., 1984; Levy *et al*., 2001) and the cyclopentenone PGs (Gilroy *et al*., 1999). Identification of these molecules indicated that resolution of inflammation is an active process coordinated by autacoids produced at the site that control immune cell responses. Introducing quantitation indices to mark resolution permitted the delineation of the *in vivo* biological actions of pro‐resolving mediators, including the lipoxins, resolvins and protectins, during acute inflammation (Bannenberg *et al*., 2005; Schwab *et al*., 2007). Detailed studies investigating mechanisms controlling the termination of inflammation also uncovered a link between the inflammation‐initiating eicosanoids PGE_**2**_ and PGD_**2**_ and lipoxin biosynthesis. These PGs are important in up‐regulating the expression of 15‐lipoxygenase type 1 (15‐LOX‐1), the initiating enzyme in the lipoxin biosynthetic pathway (Levy *et al*., 2001). Of note, inhibition of PG biosynthesis using NSAIDs is linked to a reduction in the biosynthesis of specialized pro‐resolving mediators and a delay in the termination of inflammation in experimental systems (Fukunaga *et al*., 2005). Thus, these studies demonstrated that the acute inflammatory response is a coordinated process with the initiation and termination phases being intricately linked.

These initial observations also raised the question whether novel molecules are produced during acute inflammation to promote its termination and activate reparative and regenerative responses thereby paving the way to the re‐establishment of tissue and organ function. Using a systems approach, we uncovered three novel mediator superfamilies that actively reprogramme the host immune response to halt inflammation and re‐establish organ function (Serhan *et al*., 2000; 2002; 2009; Dalli *et al*., 2013; 2014; 2015a). Given their potent biological actions, these mediators were termed as specialized pro‐resolving mediators (SPM). This superfamily is composed of mediators that are produced *via* the stereoselective conversion of essential fatty acids and include the docosahexaenoic acid derived resolvins (RvD), protectins and maresins and the eicosapentaenoic acid derived resolvins (RvE) (Serhan *et al*., 2017). The production of these mediators is regulated in a time, organ and stimulus‐dependent manner (see Dalli, 2017), and their relative concentrations to classic eicosanoids are also dependent on these factors. For example, in cerebrospinal fluids from patients with multiple sclerosis PGE_2_ and **RvD1** concentrations were present at similar concentrations (~1 pg·mL^−1^) (Pruss *et al.,* 2013). In experimental models of eye infections, LXA_**4**_ concentrations were between 2‐ and 10‐fold higher than those of PGE_2_. The production of eicosanoids and SPM is also regulated in a sex‐dependent manner where LXA_4_ concentrations in females were elevated when compared to males during experimental eye infections (Livne‐Bar *et al*., 2017), whereas RvD and RvE are elevated during experimental inflammation in humans (Rathod *et al*., 2017). The scope of the present review is to discuss the evidence underpinning the protective actions of SPM and how insights into their biological actions may provide leads on the utility of harnessing SPM as templates for the development of resolution pharmacology‐based therapeutics that would carry a lower burden of side effects.

## The identification and structure elucidation of novel immunoresolvents

### The EPA bioactive metabolome

In order to establish whether novel molecules are produced during the resolution phase that actively promote the termination of inflammation, it is essential to employ a systems approach. For this purpose, we developed metrics to measure the kinetics of cellular trafficking as well as tissue regeneration responses (Bannenberg *et al*., 2005; Schwab *et al*., 2007). Using this approach, we found that during acute, self‐limited inflammation, **eicosapentaenoic acid (EPA)** was converted in inflammatory exudates to novel mediators that potently and stereospecifically promoted the termination of inflammation and were thus coined as E‐series resolvins (RvE) (Serhan *et al*., 2000).

Investigations into the mechanisms activated by these molecules demonstrated that these mediators directly counter‐regulate the production of pro‐inflammatory mediators, including TNF‐α and inflammatory eicosanoids. In addition, the biological actions of **RvE1** at controlling leukocyte responses were found to be more potent than those of aspirin and dexamethasone. RvE1 was also found to carry potent antinociceptive actions reducing inflammatory pain by regulating both central and peripheral responses with activities of 10 ng per mouse when administered intrathecally and 285–570 pmol when administered peripherally *in vivo*. These actions were also displayed *in vitro* at concentrations as low as 3 nM (Xu *et al*., 2010; Jo *et al*., 2016; Fonseca *et al*., 2017). Of note, the antinociceptive properties of RvE1 are more potent than those exerted by the COX‐2 inhibitor **NS398** and **morphine** (Xu *et al*., 2010).

In studies assessing the role of omega‐3 supplementation in patients with arthritis, Barden and colleagues (2016) found an association between the synovial fluid concentrations of RvE2 and joint pain, where higher RvE2 concentrations were associated with lower pain scores in these patients. RvE2 is also antidepressant; i.c.v. infusions (10 ng per mouse) reduced LPS‐induced depression in mice *via* the activation of mTORC1 signalling in the medial prefrontal cortex and hippocampal dentate gyrus (Deyama *et al*., 2018). Isobe and colleagues also found that the RvE1 and RvE2 precursor 18‐hydroxy‐eicosapentaenoic acid is converted by eosinophils to a novel bioactive mediator denoted as RvE3. This mediator, at 10–100 ng per mouse, displays potent anti‐inflammatory properties characteristic of the SPM family, including the ability to regulate neutrophil recruitment (Isobe *et al*., 2012; Isobe *et al*., 2013).

### The DHA bioactive metabolome – protectins

EPA is not the only omega‐3 essential fatty acid that is converted during acute self‐limited inflammation to novel bioactive mediators. Docosahexaneoic acid is also a substrate for the formation of two SPM families produced *via* a key 17‐hydroperoxy‐docosahexaenoic acid intermediate and coined as D‐series resolvins and protectins (Serhan *et al*., 2017). In peripheral tissues, these mediators are produced by leukocytes and their biosynthesis is temporally regulated (Dalli *et al*., 2013a; Winkler *et al*., 2016). In neural tissues, the protectin biosynthetic pathway regulates both stromal and immune cell responses. In retinal pigmented cells, protectin D1 (PD1; 50 nM; referred to as NPD1 in neuronal systems) potently counteracts H_2_O_2_/TNF‐α oxidative‐stress‐triggered DNA damage. PD1 also up‐regulates the expression of several anti‐apoptotic proteins including **Bcl‐2** and **Bcl‐xL** and decreases the expression of the pro‐apoptotic factors **Bax** and **Bad** as well as the executioner caspase, **caspase 3** (Mukherjee *et al*., 2004). In cytokine‐stressed human neural cells, PD1 formation was associated with an attenuation of **amyloid‐β** secretion (Lukiw *et al*., 2005). This SPM was also reduced in the hippocampal *cornu ammonis* region 1 from patients with Alzheimer's disease but not in the thalamus or occipital lobes from the same brains. Furthermore, the expression of key enzymes in the biosynthesis of PD1, cytosolic PLA_**2**_ and 15‐LOX, was altered in the hippocampus of patients with Alzheimer's disease (Lukiw *et al*., 2005). NPD1 at 300 ng per eye also reduces the severity and incidence of stromal keratitis and corneal neovascularization following herpes simplex virus infections (Rajasagi *et al*., 2013).

Recent studies have implicated a subset of eosinophils in the biosynthesis of PD1 during the course of self‐limited inflammation. Eosinophil recruitment to inflamed loci during the resolution phase of acute inflammation correlates with an increase in PD1 production (~100 pg per exudate; Yamada *et al*., 2011). Depletion of eosinophils results in a delay in resolution responses, including an impairment of lymphatic drainage with a reduction in the appearance of phagocytes carrying engulfed zymosan in the draining lymph node and sustained numbers of polymorphonuclear leukocytes in inflamed tissues. The resolution deficit caused by eosinophil depletion was rescued by eosinophil restoration or the administration of PD1, 5 μg per mouse (Yamada *et al*., 2011). Of note, PD1 production is reduced from 2.23 ± 1.55 ng·mL^−1^ in healthy volunteers to trace concentrations in exhaled breath from asthmatics (Levy *et al*., 2007). This reduction in PD1 biosynthesis is also observed in isolated eosinophils from patients with severe asthma, suggesting a role for defective production of this SPM in disease onset and/or propagation (Miyata *et al*., 2013).

### The DHA bioactive metabolome – resolvins

The D‐series resolvins are now thought to regulate host immune responses in a number of disease settings. In *Pseudomonas aeruginosa*‐mediated lung infection **RvD1**, at 100 ng per mouse, significantly reduces P. aeruginosa titres, leukocyte infiltration and lung tissue damage (Codagnone *et al.,* 2018). In murine lung macrophages sorted during P. aeruginosa chronic infection, RvD1 regulates the expression of Toll‐like receptors (TLRs), downstream genes, as well as microRNA (miR)‐21 and 155, resulting in a reduction in inflammatory signalling. *In vitro*, RvD1 up‐regulates P. aeruginosa phagocytosis by both neutrophils and macrophages (Codagnone *et al*., 2018).

RvD2 was recently identified in skeletal muscle biopsies from humans with peripheral artery disease at concentrations of ~150 pg·g^−1^ of tissue. When this mediator was administered to mice (100 ng per mouse) during ischaemia/reperfusion, it enhanced perfusion recovery and promoted arteriogenesis (Zhang *et al*., 2016). In contrast to other strategies used for revascularization that exacerbate inflammation, RvD2 does not enhance vascular permeability, it reduces neutrophil accumulation in the damaged tissues and plasma inflammatory cytokine levels, including TNF‐α and granulocyte macrophage colony‐stimulating factor. RvD2 also increases myocyte regeneration, enhances endothelial cell migration in a Rac‐dependent manner and restores defective revascularization in diabetic mice (Zhang *et al*., 2016). In alipoliprotein E (ApoE) deficient mice, RvD2 concentrations also correlated with the signs of plaque stability, and an injection of RvD2 (100 ng per mouse) to ApoE^−/−^ mice prevented atheroprogression, reduced macrophage recruitment, increased fibrous cap thickness and smooth muscle cell numbers (Viola *et al*., 2016).

Additionally, RvD3 displays potent tissue protective actions. RvD3 was identified in uninjured lungs, suggesting that it plays a functional role in regulating tissue resolution tone (Colby *et al*., 2016). The concentrations of this mediator are rapidly up‐regulated following hydrochloric acid‐initiated injury from ~22 to ~75 pg per lung. Administration of the metabolically stable endogenous isomer of RvD3, 17R‐RvD3 (10 ng per mouse), affords significant tissue protection during hydrochloric acid‐initiated injury, reducing alveolar wall thickening, lung oedema and leukocyte infiltration (Colby *et al*., 2016). This mediator also increases lung epithelial cell proliferation, bronchoalveolar lavage fluid levels and keratinocyte growth factor promoting cutaneous re‐epithelialization (Colby *et al*., 2016).

**Figure 1:**
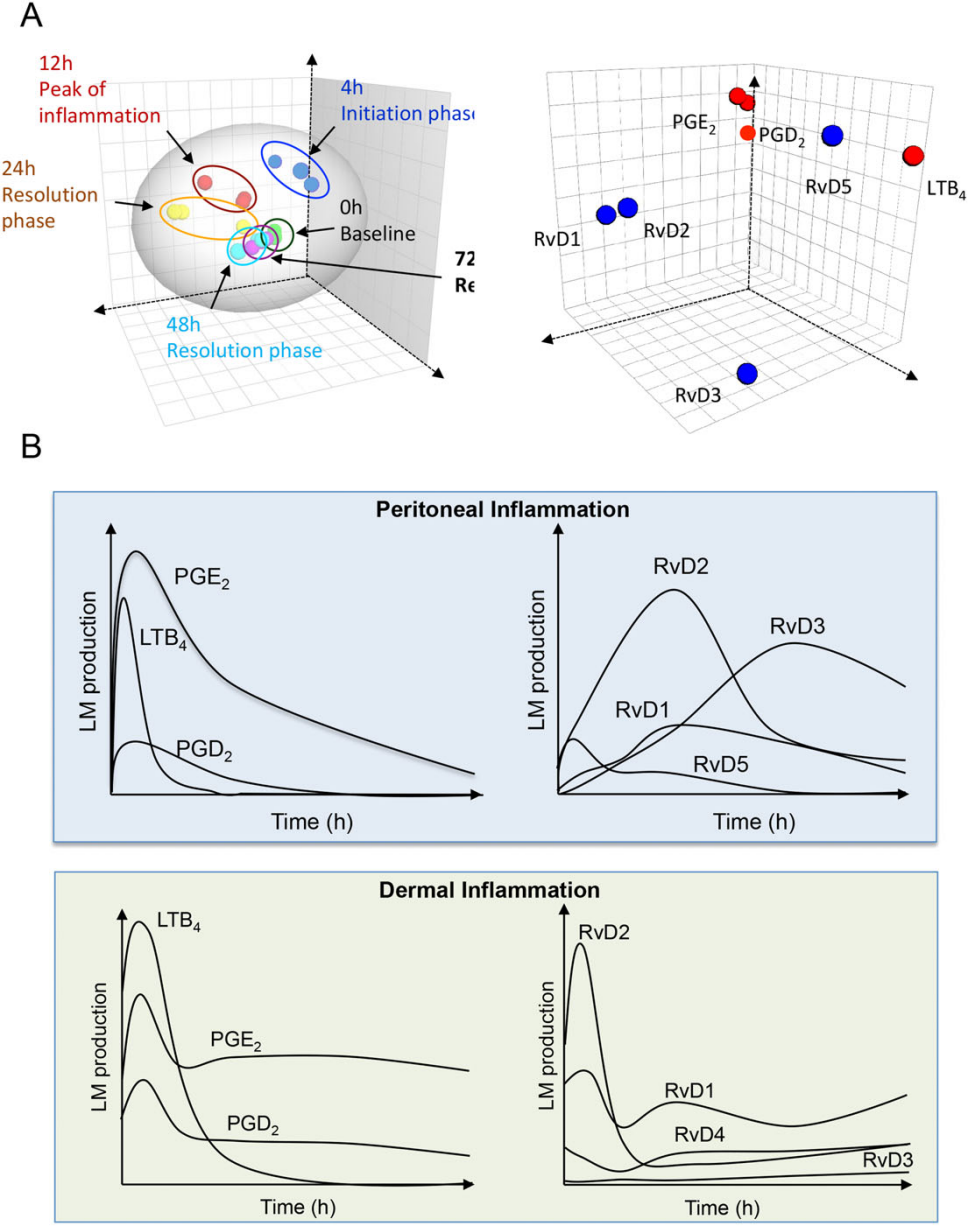
Temporal, tissue and stimulus specific SPM regulation during sterile and infectious inflammation. (A) Mice were challenged with 1 μg of zymosan; inflammatory exudates were collected at the indicated intervals and lipid mediators quantified as in Dalli *et al*. (2013a). Results were analysed using multivariate analysis. (A, left panel) Depicts the temporal shift in lipid mediator clusters during the course of initiation and resolution. (A, right panel) Denotes the contribution of each mediator to the temporal clustering. (B, top panel) Mice were challenged and lipid mediators were identified and quantified. Panels are illustrations of results presented in Dalli *et al*. (2013a). (B; bottom panel) Mice were challenged with Staphylococcus aureus and lipid mediators identified and quantified. Panels are illustrations of results presented in Winkler *et al*. (2016).

In murine tissues infected with Staphylococcus aureus, recent studies found that RvD4 persists late into the resolution phase at concentrations of 0.75 pg per exudate (Winkler *et al*., 2016). Administration of this mediator (200 ng per mouse) during S. aureus infection reduces neutrophilic infiltration, increases bacterial clearance and restores resolution responses. RvD4 also enhanced the uptake of apoptotic neutrophils by human dermal fibroblasts at concentrations as low as 0.1 nM (Winkler *et al*., 2016). RvD5 displays potent anti‐bacterial actions, up‐regulating neutrophil and macrophage phagocytosis of bacteria in a dose‐ and receptor‐dependent manner (Chiang *et al*., 2012). Of note, RvD biosynthesis in tissues is regulated in both a stimulus‐ and tissue‐dependent manner, thereby demonstrating that these mediators exert distinct biological actions during self‐limited inflammation (Figure 1) (Chiang *et al*., 2012; Dalli *et al*., 2013a; Winkler *et al*., 2016).

### The DHA bioactive metabolome – maresins

Macrophages are central players in regulating the host immune response. We recently found that these cells also produce a novel family of mediators termed maresins (*ma*crophage mediators in *re*solving *in*flammation) (Serhan *et al*., 2009). The biosynthesis of this family of mediators is initiated by lipoxygenation and subsequent epoxidation of DHA to yield 13S,14S‐epoxy‐MaR (13S,14S‐eMaR). This product regulates the production of the leukocyte chemoattractant LTB_4_ *via* the direct inactivation of the LTA_4_ hydrolase (Dalli *et al*., 2013b). 13S,14S‐eMaR (10 nM) also increases the expression of markers associated with an alternatively activated phenotype in human macrophages, including CD163 and CD206, and down‐regulating the expression of markers linked with a classically activated phenotype including CD54 (Dalli *et al*., 2013b). In addition to directly regulating aspects of the immune response, 13S,14S‐eMaR is also converted to MaR1 and MaR2 *via* enzyme‐mediated hydrolysis (Serhan *et al*., 2009; Dalli *et al*., 2013b; Deng *et al*., 2014).

MaR1, the first member of this family identified, is antinociceptive regulating the activation of **TRPV1** by capsaicin at concentrations as low as 0.5 ng·mL^−1^. Administration of MaR1 at 10 ng per mouse also inhibits chemotherapy‐elicited neuropathic pain (Serhan *et al*., 2012). MaR1 exerts protective actions on hepatocytes, reducing lipotoxicity‐induced apoptosis by activating the unfolded protein response pro‐survival mechanisms and limiting the up‐regulation of pro‐apoptotic pathways (Rius *et al*., 2017). This macrophage‐derived mediator regulates the expression of miRNA targeting both protein folding and apoptosis and enhances the phagocytic capacity of Kupffer cells (Rius *et al*., 2017).

Recent studies also demonstrate that MaR1 is produced by circulating neutrophil–platelet aggregates. During these heterotypic cell aggregates, DHA is converted to 13S,14S‐eMaR by the platelet 12‐LOX. This intermediate is then donated to neutrophils that, *via* enzyme‐mediated hydrolysis, produce MaR1 (Abdulnour *et al*., 2014). The production of MaR1 by these heterotypic cell aggregates in the vasculature is a protective mechanism engaged to limit tissue damage following acid‐induced lung injury and results in a reduction of activated neutrophil recruitment into the damaged tissues (Abdulnour *et al*., 2014). At the site of infectious inflammation, MaR1 is subjected to further metabolism by exudate leukocytes. Macrophages convert this mediator to 14‐oxo‐MaR1, which displays blunted biological actions in comparison to the parent mediator. Neutrophils convert MaR1 to 22‐OH‐MaR1 that retains the potent biological actions of the parent SPM, including its ability to regulate the activation of the LTB_4_ receptor (BLT_**1**_ **receptor**) by its cognate ligand (Colas *et al*., 2016). In the CNS, MaR1 also displays reparative actions, where MaR1 (1 μg per mouse, i.v.) administration to animals subjected to spinal cord injury improved locomotor recovery and reduced secondary injury progression (Francos‐Quijorna *et al*., 2017).

### The DHA bioactive metabolome – sulfido conjugated SPM

In recent studies, we found that 13S,14S‐eMaR is also an intermediate in the biosynthesis of a novel family of peptide‐lipid conjugated molecules termed maresin conjugates in tissue regeneration (MCTR) (Dalli *et al*., 2014). This family of mediators is evolutionary conserved being identified in planaria, mice and humans. Furthermore, in each of these species, MCTR displays potent tissue protective and tissue regenerative actions. During murine infections, MCTRs are produced late within the resolution phase, coinciding with the turning on of reparative and regenerative responses (Dalli *et al*., 2014). Administration of MCTRs (100 nM) to planaria following surgical injury leads to the up‐regulation of genes involved in tissue regeneration. In mice following ischaemia–reperfusion mediated injury, MCTR1 and MCTR2 (50 ng per mouse each) up‐regulate the expression of proteins involved in lung repair and regeneration, including Ki67 and **R‐spondin 3** (Dalli *et al*., 2014). Identification of the MCTRs also paved the way to the identification of two novel peptide lipid conjugated mediator families termed as protectin conjugates in tissue regeneration (PCTR) and resolvin conjugates in tissue regeneration (RCTR) (Dalli *et al*., 2015b). These mediators display tissue regenerative and leukocyte directed actions controlling a host's response to the Gram‐negative bacterium Escherichia coli (Dalli *et al*., 2015b). PCTR1 production in the murine peritoneum is under neural control (Dalli *et al*., 2017). This mediator is central in maintaining tissue resolution tone *via* regulating tissue resident macrophage phenotype and function. Disruption of the vagus nerve or inhibiting 15‐LOX, the initiating enzyme in the PCTR biosynthetic pathway, perturbs PCTR production leading to impaired host responses to E. coli infections, including delayed resolution of self‐limited inflammation and a reduction in the ability of recruited leukocytes to phagocytose and kill bacteria (Dalli *et al*., 2017).

### The n‐3 docosapentaenoic acid bioactive metabolome

**Figure 2:**
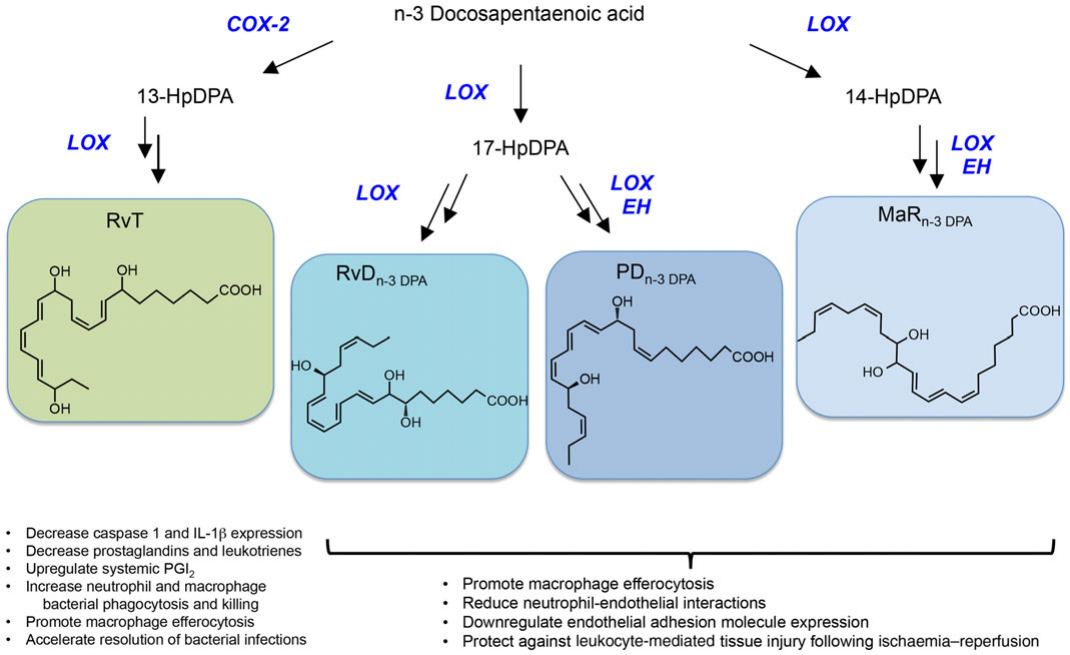
Novel immunoresolvents biosynthesized from n‐3 docosapentaenoic. In the vasculature, n‐3 docosapentaenoic acid is the substrate for conversion, by endothelial COX‐2, to 13‐HDPA that is then donated to neutrophils and converted to RvT that display potent protective actions in infectious inflammation (Dalli *et al*., 2015a). The n‐3 docosapentaenoic is also converted by leukocytes to 17‐HpDHA that is a precursor to RvD_n‐3 DPA_ and PD_n‐3 DPA_. Conversion of n‐3 DPA *via* 14‐lipoxygenation yields 14‐HpDPA that is converted to MaR_n‐3 DPA_. Each of these mediator families displays potent leukocyte‐directed and host‐protective actions (Dalli *et al*., 2013; Gobbetti *et al*., 2017).

We recently found that n‐3 docosapentaenoic acid (DPA) was not simply a precursor in the biosynthesis of DHA from EPA. This essential fatty acid is converted by leukocytes to several mediator families that are congeners to those produced when DHA is the substrate (Figure 2) (Dalli *et al*., 2013). In murine plasma and inflammatory exudates following ischaemia–reperfusion injury, we found that n‐3 DPA is converted to the D‐series resolvins, RvD1_n‐3 DPA_, RvD2_n‐3 DPA_ and RvD5_n‐3 DPA_ as well as PD1_n‐3 DPA_ with concentrations at the site of inflammation for these SPM ranging between 20 and 100 pg per exudate (Dalli *et al*., 2013). These new mediators each display potent leukocyte‐directed properties, reducing neutrophil recruitment during acute peritonitis and leukocyte‐mediated lung damage during ischaemia–reperfusion (Dalli *et al*., 2013; Aursnes *et al*., 2014). Human leukocytes, in addition to producing RvD_n‐3 DPA_ and PD_n‐3 DPA_, also convert n‐3 DPA to MaR_n‐3 DPA_, which displays leukocyte and endothelial directed actions reducing the expression of CD54 in TNF‐α activated endothelial cells (Dalli *et al*., 2013; Tungen *et al*., 2014). The biosynthesis of RvD5_n‐3 DPA_ and PD1_n‐3 DPA_ is disrupted in colonic biopsies from patients with inflammatory bowel disease as well as in mice given dextran sodium sulphate (DSS) (Gobbetti *et al*., 2017). Administration of either of these mediators provided significant protection against DSS‐initiated colon inflammation. Furthermore, increasing the endogenous production of these mediators in mice during DSS‐initiated colitis also led to a significant reduction in tissue damage (Gobbetti *et al*., 2017). In this context, recent studies in healthy volunteers found that supplementation with n‐3 DPA increased plasma RvD5_n‐3 DPA_ concentrations (Markworth *et al*., 2016). Using a lipid mediator profiling approach, we found that in humans systemic concentrations of RvD_n‐3 DPA_ diurnally regulated and their concentrations correlate with markers of both platelet and leukocyte activation including CD11b and CD62P (Colas *et al*., 2018).

In the vasculature during ongoing inflammation, n‐3 DPA is also converted to the 13‐series resolvins (RvT) (Dalli *et al*., 2015a). The biosynthesis of these mediators is initiated by endothelial **COX‐2** that converts the essential fatty acid to 13‐(R)‐hydroxyperoxy‐7Z,10Z,13R,14E,16Z,19Z‐docosapentaenoic acid (13‐HpDPA). This or its reduced form, namely, 13‐HDPA, are then donated to neutrophils for transcellular biosynthesis to produce RvT (Dalli *et al*., 2015a; Primdahl *et al*., 2016). These mediators are produced during the early stages of self‐limited bacterial infections with plasma concentrations reaching concentrations of ~30–50 pg·mL^−1^. These mediators regulate the ability of leukocytes to uptake and kill bacteria. RvT also down‐regulate the activation of the NACHT, LRR and PYD domain‐containing protein 3 (**NALP3**) inflammasome in murine and human macrophages, reducing the expression of **caspase 1** and the production of **IL‐1β** as well as the release of LDH, a hallmark of pyroptosis (Dalli *et al*., 2015a). In addition, RvTs are also important in mediating the biological actions of atorvastatin and pravastatin (Dalli *et al*., 2015a; Walker *et al*., 2017). Administration of these statins regulates COX‐2 activity by up‐regulating the **NOS**‐mediated nitrosylation of the enzyme, increasing its catalytic turnover and 13‐HDPA formation (Dalli *et al*., 2015a). Inhibition of either NOS or COX‐2 using specific pharmacological inhibitors led to a reversal of the protective actions of atorvastatin and pravastatin in both infectious inflammation and rheumatoid arthritis (Dalli *et al*., 2015a; Walker *et al*., 2017).

## How do the SPM exert their biological actions?

### Agonists of GPCRs

**Figure 3:**
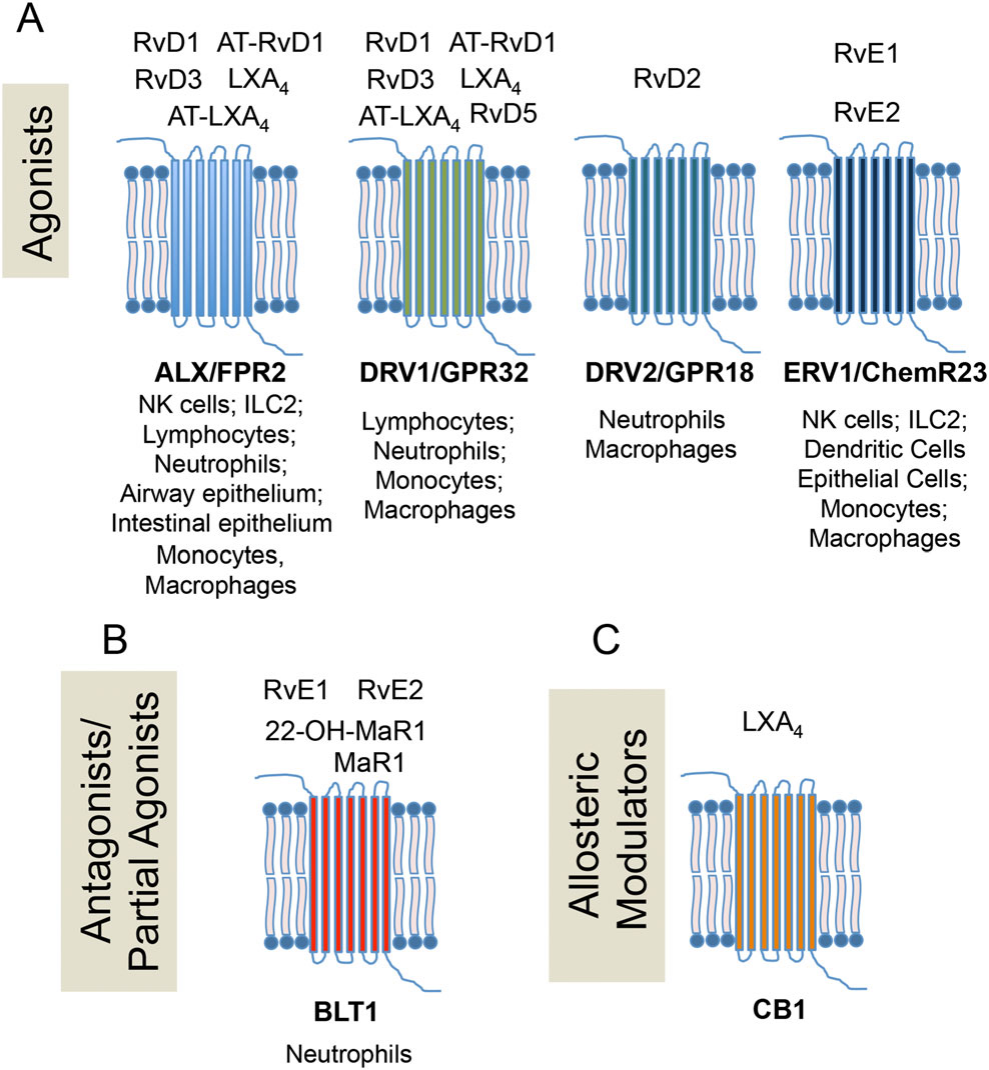
Distinct interactions between SPM and GPCRs in controlling host responses. (A) Depicts the receptors activated by select pro‐resolving mediators and the target cells in which these receptors mediate the actions of their cognate SPM. (B,C) Depicts the receptors at which each SPM acts as a (B) antagonists/partial agonist or (C) allosteric modulator.

The biological actions of SPM are stereoselective and are mediated *via* the activation of cognate receptors and signalling pathways. The first of these receptors to be identified was the LXA_4_ receptor, which up until then was thought to be a low affinity receptor for endogenous formylated peptides (Fiore *et al*., 1994). The LXA_4_ receptor (ALX) is part of the GPCR family (Figure 3). Its activation by cognate endogenous ligands, including LXA_4_ and the pro‐resolving protein annexin A1, displays a characteristic bell shape, where at either end of the dose range, the ability of the ligand to activate the receptor is significantly reduced (Hayhoe *et al*., 2006; Rovira *et al*., 2010). Of note, ALX receptor activation by its pro‐resolving ligands leads to distinct downstream signalling pathways that are cell specific. For example, LXA_4_ increases intracellular calcium levels in conjunctival goblet cells *via* the ALX receptor (Hodges *et al*., 2016); whereas activation of this receptor by LXA_4_ does not elicit a calcium response in human neutrophils (Fiore and Serhan, 1995). Studies conducted by Cooray and colleagues demonstrate that the ALX receptor on cell membranes is expressed as a homo‐ or heterodimer with the formyl peptide receptor **FPR1** or **FPR3** (the two other family members) and operates in a ligand‐biased fashion. Furthermore, signalling pathways activated by these different dimerization states are distinct (Cooray *et al*., 2013). This process of dimerization is at least in part influenced by the ligands themselves whereby annexin A1 promotes homodimerization and the activation of p38/MAPK‐activated protein kinase/heat shock protein 27 signalling and IL‐10 up‐regulation (Cooray *et al*., 2013). On the other hand, the pro‐inflammatory acute phase protein serum amyloid protein A did not lead to receptor homodimerization. These findings shed light on how one receptor can mediate the biological actions of functionally distinct molecules (Cooray *et al*., 2013). In addition to mediating the biological actions of LXA_4_ and its aspirin triggered epimer (Ortiz‐Munoz *et al*., 2014; Wang *et al*., 2014; Romano *et al*., 2015), the ALX receptor also mediates the actions of the D‐series resolvins RvD1, RvD3 and their aspiring trigger epimers (Krishnamoorthy *et al*., 2012; Arnardottir *et al*., 2016; de Oliveira *et al*., 2017; Mottola *et al*., 2017; Serhan *et al*., 2017).

Activation of the orphan receptor **GPR32** by RvD1 leads to the regulation of a number of miRNA involved in the orchestration of acute inflammation, including miR‐146b, miR‐208a and miR‐219 (Krishnamoorthy *et al*., 2012; Serhan *et al*., 2017). This receptor also mediates the biological actions of RvD5 in the context of bacterial infections, whereby its activation by RvD5 leads to enhanced bacterial phagocytosis in human macrophages and a down‐regulation of several pro‐inflammatory genes, including NF‐κB and TNF‐α (Chiang *et al*., 2012). In lung cancer cells, GPR32 also mediates the biological actions of RvD1 in preventing **TGFβ1**‐induced epithelial‐mesenchymal‐transition (Lee *et al*., 2013). Additionally, this receptor is involved in regulating macrophage phenotype and function, whereby it mediates RvD1‐initiated regulation of pro‐inflammatory cytokines IL‐1β and IL‐8. GPR32 is also involved in mediating the inhibitory actions of RvD1 on macrophage chemotaxis towards chemerin, fMLF and **monocyte chemoattractant protein‐1 (also known as CCL2)** (Schmid *et al*., 2016).

The biological actions of the E‐series resolvins, RvE1 and RvE2, are mediated by **chemerin receptor 2** /resolvin E1 (chemerin_**1**_) receptor (ERV1; Arita *et al*., 2005; Oh *et al*., 2012). RvE1 signalling *via* the chemerin_1_ receptor (ERV1) leads to the activation of PI3K and ERK resulting in the phosphorylation of both Akt and the ribosomal protein S6 (Arita *et al*., 2005). The expression of this receptor in human diabetic patients is up‐regulated. Of note, despite increased expression of the RvE receptor in diabetic neutrophils, the biological actions of RvE1 in these patients are blunted suggesting that impaired/defective signalling of this receptor in diabetes may be a component in the ethiopathogenis of the disease (Freire *et al*., 2017). The expression of chemerin_1_ receptors (ERV1) in human monocyte‐derived macrophages was recently shown to be regulated by **LPS** and **IFN‐γ**, where incubation of monocyte‐derived macrophages with these inflammatory signals activates the promoter of these receptors (Herova *et al*., 2015).

GPR18 (resolvin D2 receptor; DRV2) is a receptor for RvD2. The binding of the agonist to its receptor leads to an increase in intracellular cAMP as well as CREB, ERK1/2 and STAT3 phosphorylation. Loss of this receptor in transgenic animals is associated with delayed resolution response to both sterile and infectious inflammatory insults (Chiang *et al*., 2015; Zhang *et al*., 2016) as well as increased mortality during polymicrobial sepsis (Chiang *et al*., 2017). Silencing of GPR18 (DRV2) on human macrophages also leads to a loss in their ability to clear bacteria and apoptotic cells, two key resolution responses, thereby underscoring the role of this RvD2‐GPR18 axis in restabilising tissue function following an inflammatory insult (Chiang *et al*., 2015; 2017).

### SPM as allosteric modulators and antagonists

In addition to activating cognate receptors, SPM also regulate the activity of receptors for other endogenous mediators. LXA_4_ was recently found to be an endogenous allosteric modulator of the cannabinoid CB_1_ receptor (Pamplona *et al*., 2012). This SPM enhanced the affinity of anandamide at the CB_1_ receptor potentiating its biological actions without competing for the orthosteric binding site of the CB_1_ receptor and altering endocannabinoid metabolism (Figure 3). In addition, LXA_4_ displayed protective actions against β‐amyloid (1–40)‐induced spatial memory impairment, actions that were CB_1_ receptor‐dependent (Pamplona *et al*., 2012).

Some SPM also act as antagonists to receptors of pro‐inflammatory eicosanoids. RvE1, MaR1 and its further metabolite 22‐OH‐MaR1 are all competitive antagonists of BLT1 receptors (Figure 3) inhibiting the signalling and biological actions of the potent leukocyte chemoattractant LTB_4_ (Arita *et al*., 2005; El Kebir *et al*., 2012; Oh *et al*., 2012; Colas *et al*., 2016). In human neutrophils, BLT1 antagonism enhances neutrophil apoptosis (El Kebir *et al*., 2012). Of note, during self‐limiting infectious inflammation, MaR1 concentrations reach a maximum early on in the course of the inflammatory process, concomitant with a rise in LTB_4_ (Colas *et al*., 2016). This suggests that, MaR1 production may play an important role in antagonizing the actions of LTB_4_, thereby limiting neutrophil recruitment. These results also indicate that disruptions in the biosynthesis of this mediator may lead to delayed resolution of responses. Together, these findings highlight the utility of SPM as templates for the development of novel receptor modulators.

## Harnessing SPM biology towards resolution pharmacology

SPM display potent biological actions without the side effects displayed by traditional anti‐inflammatories. The reason for this lack of unwanted side effects may be attributable to the mechanisms activated by SPM, which are distinct from those regulated by anti‐inflammatories. Indeed, SPM do not completely abolish the production and actions of molecules and cells considered to be inflammatory. Instead, these mediators reprogramme both the immune response and stromal cells limiting the production of inflammatory molecules and up‐regulate the expression of protective mediators (Mukherjee *et al*., 2004; Pamplona *et al*., 2012; Lee *et al*., 2013; Miyata *et al*., 2013; Dalli *et al*., 2014; Herova *et al*., 2015; Colby *et al*., 2016; Codagnone *et al*., 2018; de Oliveira *et al*., 2017). This reprogramming of the immune system is also linked to the apparent lack of immunosuppressive action. Thus, prompted by these observations, several studies have explored the potential of harnessing these actions to control excessive inflammation characteristic of many diseases that afflict modern societies (Takano *et al*., 1997; Leonard *et al*., 2002; Mitchell *et al*., 2002; Guilford *et al*., 2004).

From these efforts, several generations of analogues and mimetics are now developed (see Table 1) with the aim of obtaining molecules with enhanced pharmacokinetics and pharmacodynamics that are also amenable to scaled‐up synthesis (Takano *et al*., 1997; Leonard *et al*., 2002; Mitchell *et al*., 2002; Guilford *et al*., 2004). The first of these developed was the lipoxin analogues that retain the biological actions of their parent molecules but with enhanced stability. One of these analogues, a benzo‐LXA_4_, displays potent host protective actions that include the ability to regulate neutrophil responses and promote tissue repair and regeneration in periodontal disease (Van Dyke *et al*., 2015). These observations have now been extended to humans where the protective actions of this analogue are being investigated in the context of human periodontal disease (ClinicalTrials.gov Identifier: NCT02342691). A later generation analogue of RvE1 displays potent protective actions in dry eye syndrome in humans. In a phase 2 trial, this RvE1 analogue significantly reduced disease incidence, controlling dry eye‐related inflammation, findings that are under further investigation in a phase 3 trial (Wire, 2009.).

**Table 1:** Biological actions of SPM analogues

Analogue	Biological action	Dose	Reference
Lipoxin analogues			
15(R/S)‐methyl‐LXA_4_	Inhibits vascular permeability change and PMN infiltration Stimulates phagocytosis of apoptotic PMN by macrophages *in vivo*	3–130 nM 2.5–10 μg·kg^−1^	(Takano *et al*., 1998) (Mitchell *et al*., 2002)
16‐phenoxy‐LXA_4_	Inhibits neutrophil infiltration in response to LTB_4_	240 nM	(Takano *et al*., 1997)
15‐epi,16‐phenoxy‐LXA_4_	Inhibits neutrophil infiltration in response to LTB_4_	240 nM	(Takano *et al*., 1997)
16‐parafluoro‐phenoxy‐LXA_4_	Inhibits PMN infiltration	26 nM	(Takano *et al*., 1998)
5(S)‐methyl‐LXB_4_	Inhibits PMN infiltration	26 nM	(Takano *et al*., 1998)
15‐epi, 16‐para‐fluorophenoxy‐LXA_4_‐methyl ester	Tissue protection and reduced neutrophil infiltration during kidney ischaemia–reperfusion. Reduces tissue IL‐1β, IL‐6, and GRO1 mRNA levels Prevents airway hyper‐responsiveness to methacholine Reduces eosinophils and lymphocytes infiltration Protects against vascular injury Reduces skin inflammation Inhibits human neutrophil chemotaxis Reduces colon inflammation Inhibits VEGF‐induced EC migration and proliferation	15 μg per mouse 10 μg per mouse 100–1000 μg·cm^−2^ 0.1 nM–1 μM 10 μg·mL^−1^ (p.o) 1–100 nM	(Leonard *et al*., 2002) (Levy *et al*., 2002) (Schottelius *et al*., 2002) (Gewirtz *et al*., 2002) (Cezar‐de‐Mello *et al*., 2008)
o‐[9,12]‐benzo‐ω6‐epi‐lipoxin A_4_	Reducec leukocyte infiltration into the tempo mandibular joint following CFA administration Reduces neutrophil recruitment in response to zymosan Promotes regeneration of hard and soft tissues irreversibly lost to periodontitis in the Hanford miniature pig	10 ng per mouse 100 ng per mouse 1 μg per site	(Norling *et al*., 2011) (Van Dyke *et al*., 2015)
D‐series resolvin analogues			
7R/S methyl RvD1 methyl ester	Reduces DC expression of MHC II, CD40 and IL‐12 following LPS stimulation Reduces allosensitization during corneal transplant and enhances graft survival and angiogenesis	100 μg per mouse	(Hua *et al*., 2014)
Benzo‐diacetylenic‐17R‐RvD1‐methyl ester	Shortens the resolution interval, Ri, during E. coli peritonitis Reduces ischaemia‐reperfusion‐initiated second organ injury	100 ng per mouse 1 μg per mouse	(Orr *et al*., 2015)
E‐series resolvin analogues			
RX‐10045	Substrate/inhibitor for efflux transporters multidrug resistance‐associated protein, breast cancer‐resistant protein and organic cation transporter‐1 Reduces corneal opacity after haze‐generating after opacity‐generating high correction photorefractive keratectomy Reduction from baseline in controlled adverse environment‐induced staining of the central cornea Improvement in human dry eye disease symptoms, including dryness, stinging, burning, grittiness and ocular discomfort	50–300 μM 0.01% solution 0.05–0.1% nanomicellar solution	(Cholkar *et al*., 2015) (Torricelli *et al*., 2014) (Wire, 2009)
α‐cyclopropane resolvin E2	Reduces the number of exudate leukocytes in response to *Propionibacterium acnes* infection	300 fg–3 ng per mouse	(Fukuda *et al*., 2016)
β‐cyclopropane resolvin E2	Reduces the number of exudate leukocytes in response to *P. acnes* infection	300 fg–3 ng per mouse	(Fukuda *et al*., 2016)

Additionally, recent studies have demonstrated the protective actions of analogues of RvD1 (Orr *et al*., 2015) and RvE2 (Fukuda *et al*., 2016). The RvD1 analogues retain the ability to activate GPR32 (DRV1) as well as the tissue protective actions of the parent SPM (Orr *et al*., 2015). Enhanced biological actions are also displayed the cyclopropane congeners of RvE2 (Fukuda *et al*., 2016), thereby supporting a role for these analogues as novel therapeutics.

## Conclusion

The appreciation that resolution of inflammation is an active process and SPM are central in controlling both cellular trafficking and responses has opened up new and exciting horizons for the development of new therapeutics. Given that resolution pharmacology‐based medicines will harness endogenous reparative responses, it is anticipated that they will be burdened with lower side effects since they will not interfere with natural host immunity, potentially also increasing patient compliance. Furthermore, these findings shed new light on the roles of omega‐3 essential fatty acids in the control of acute inflammation. The protective actions observed in many clinical studies using these fatty acids are not simply due to the inhibition of inflammatory eicosanoid formation by competing for the biosynthetic enzymes. Indeed, these substrates are precursors to structurally unique molecules, the SPM, that carry potent tissue protective actions. Thus, these mediators may represent novel markers to determine the pharmacodynamics and pharmacokinetics of omega‐3 supplements and their ability to influence the host response in both healthy people as well as patients with inflammatory diseases. While the evidence in humans for the utility of this approach is still being investigated, results from animal and initial human experiments are encouraging (Dalli *et al*., 2013; Arnardottir *et al*., 2016; Barden *et al*., 2016; Markworth *et al*., 2016; Gobbetti *et al*., 2017). Therefore, resolution‐based therapeutics provide an exciting new paradigm for personalized medicine where supplementation can be utilized to maintain/boost endogenous SPM levels to preserve tissue resolution tone and health. Whereas in disease settings, SPM‐based drugs may be useful in regulating host responses to both local and systemic inflammation. In this context, nanomedicines enriched in SPM or their analogues/mimetics (Norling *et al*., 2011) may provide a tractable system for targeted tissue delivery that can control both inflammation and promote tissue repair and regeneration (Van Dyke *et al*., 2015). Hence, resolution pharmacology could provide the basis for reprograming host immunity in order to expedite microbial clearance, limit collateral tissue damage and stimulate tissue regeneration.

### Nomenclature of targets and ligands

Key protein targets and ligands in this article are hyperlinked to corresponding entries in http://www.guidetopharmacology.org, the common portal for data from the IUPHAR/BPS Guide to PHARMACOLOGY (Harding *et al*., 2018) and are permanently archived in the Concise Guide to PHARMACOLOGY 2017/18 (Alexander *et al*., 2017a,b,c,d).

## Conflict of interest

The authors declare no financial conflicts of interest.

## References

[bph14336-bib-0001] Abdulnour RE , Dalli J , Colby JK , Krishnamoorthy N , Timmons JY , Tan SH *et al* (2014). Maresin 1 biosynthesis during platelet‐neutrophil interactions is organ‐protective. Proc Natl Acad Sci U S A 111: 16526–16531. 25369934 10.1073/pnas.1407123111 PMC4246348

[bph14336-bib-0002] Alexander SPH , Christopoulos A , Davenport AP , Kelly E , Marrion NV , Peters JA *et al* (2017a). The Concise Guide to PHARMACOLOGY 2017/18: G protein‐coupled receptors. Br J Pharmacol 174: S17–S129. 29055040 10.1111/bph.13878 PMC5650667

[bph14336-bib-0003] Alexander SPH , Fabbro D , Kelly E , Marrion NV , Peters JA , Faccenda E *et al* (2017b). The Concise Guide to PHARMACOLOGY 2017/18: Enzymes. Br J Pharmacol 174: S272–S359. 29055034 10.1111/bph.13877 PMC5650666

[bph14336-bib-0004] Alexander SPH , Fabbro D , Kelly E , Marrion NV , Peters JA , Faccenda E *et al* (2017c). The Concise Guide to PHARMACOLOGY 2017/18: Catalytic receptors. Br J Pharmacol 174: S225–S271. 29055036 10.1111/bph.13876 PMC5650661

[bph14336-bib-0005] Alexander SPH , Kelly E , Marrion NV , Peters JA , Faccenda E , Harding SD *et al* (2017d). The Concise Guide to PHARMACOLOGY 2017/18: Overview. Br J Pharmacol 174: S1–S16. 29055037 10.1111/bph.13882 PMC5650665

[bph14336-bib-0006] Arita M , Bianchini F , Aliberti J , Sher A , Chiang N , Hong S *et al* (2005). Stereochemical assignment, antiinflammatory properties, and receptor for the omega‐3 lipid mediator resolvin E1. J Exp Med 201: 713–722. 15753205 10.1084/jem.20042031 PMC2212834

[bph14336-bib-0007] Arnardottir HH , Dalli J , Norling LV , Colas RA , Perretti M , Serhan CN (2016). Resolvin D3 is dysregulated in arthritis and reduces arthritic inflammation. J Immunol 197: 2362–2368. 27534559 10.4049/jimmunol.1502268 PMC5011006

[bph14336-bib-0008] Aursnes M , Tungen JE , Vik A , Colas R , Cheng CY , Dalli J *et al* (2014). Total synthesis of the lipid mediator PD1n‐3 DPA: configurational assignments and anti‐inflammatory and pro‐resolving actions. J Nat Prod 77: 910–916. 24576195 10.1021/np4009865 PMC4000582

[bph14336-bib-0009] Bannenberg GL , Chiang N , Ariel A , Arita M , Tjonahen E , Gotlinger KH *et al* (2005). Molecular circuits of resolution: formation and actions of resolvins and protectins. J Immunol 174: 4345–4355. 15778399 10.4049/jimmunol.174.7.4345

[bph14336-bib-0010] Barden AE , Moghaddami M , Mas E , Phillips M , Cleland LG , Mori TA (2016). Specialised pro‐resolving mediators of inflammation in inflammatory arthritis. Prostaglandins Leukot Essent Fatty Acids 107: 24–29. 27033423 10.1016/j.plefa.2016.03.004

[bph14336-bib-0011] Cezar‐de‐Mello PF , Vieira AM , Nascimento‐Silva V , Villela CG , Barja‐Fidalgo C , Fierro IM (2008). ATL‐1, an analogue of aspirin‐triggered lipoxin A4, is a potent inhibitor of several steps in angiogenesis induced by vascular endothelial growth factor. Br J Pharmacol 153: 956–965. 18193074 10.1038/sj.bjp.0707650 PMC2267264

[bph14336-bib-0012] Chiang N , Dalli J , Colas RA , Serhan CN (2015). Identification of resolvin D2 receptor mediating resolution of infections and organ protection. J Exp Med 212: 1203–1217. 26195725 10.1084/jem.20150225 PMC4516788

[bph14336-bib-0013] Chiang N , de la Rosa X , Libreros S , Serhan CN (2017). Novel resolvin D2 receptor axis in infectious inflammation. J Immunol 198: 842–851. 27994074 10.4049/jimmunol.1601650 PMC5225078

[bph14336-bib-0014] Chiang N , Fredman G , Backhed F , Oh SF , Vickery T , Schmidt BA *et al* (2012). Infection regulates pro‐resolving mediators that lower antibiotic requirements. Nature 484: 524–528. 22538616 10.1038/nature11042 PMC3340015

[bph14336-bib-0015] Cholkar K , Trinh HM , Vadlapudi AD , Wang Z , Pal D , Mitra AK (2015). Interaction studies of resolvin E1 analog (RX‐10045) with efflux transporters. J Ocul Pharmacol Ther 31: 248–255. 25844889 10.1089/jop.2014.0144 PMC4426295

[bph14336-bib-0016] Codagnone M , Cianci E , Lamolinara A , Mari VC , Nespoli A , Isopi E *et al* (2018). Resolvin D1 enhances the resolution of lung inflammation caused by long‐term *Pseudomonas aeruginosa* infection. Mucosal Immunol 11: 35–49. 28422188 10.1038/mi.2017.36

[bph14336-bib-0017] Colas RA , Dalli J , Chiang N , Vlasakov I , Sanger JM , Riley IR *et al* (2016). Identification and actions of the maresin 1 metabolome in infectious inflammation. J Immunol 197: 4444–4452. 27799313 10.4049/jimmunol.1600837 PMC5127279

[bph14336-bib-0018] Colas RA , Souza PR , Walker ME , Burton M , Marques RM , Zasłona Z Curtis AM *et al* (2018). Impaired production and diurnal regulation of vascular RvD_n‐3 DPA_ increase systemic inflammation and cardiovascular disease. Circ Res 122: 855–863. 29437834 10.1161/CIRCRESAHA.117.312472 PMC5924694

[bph14336-bib-0019] Colby JK , Abdulnour RE , Sham HP , Dalli J , Colas RA , Winkler JW *et al* (2016). Resolvin D3 and aspirin‐triggered resolvin D3 are protective for injured epithelia. Am J Pathol 186: 1801–1813. 27171898 10.1016/j.ajpath.2016.03.011 PMC4929400

[bph14336-bib-0020] Cooray SN , Gobbetti T , Montero‐Melendez T , McArthur S , Thompson D , Clark AJ *et al* (2013). Ligand‐specific conformational change of the G‐protein‐coupled receptor ALX/FPR2 determines proresolving functional responses. Proc Natl Acad Sci U S A 110: 18232–18237. 24108355 10.1073/pnas.1308253110 PMC3831442

[bph14336-bib-0021] Dalli J (2017). Does promoting resolution instead of inhibiting inflammation represent the new paradigm in treating infections? Mol Aspects Med 58: 12–20. 28365269 10.1016/j.mam.2017.03.007

[bph14336-bib-0022] Dalli J , Chiang N , Serhan CN (2014). Identification of 14‐series sulfido‐conjugated mediators that promote resolution of infection and organ protection. Proc Natl Acad Sci U S A 111: E4753–E4761. 25324525 10.1073/pnas.1415006111 PMC4226123

[bph14336-bib-0023] Dalli J , Chiang N , Serhan CN (2015a). Elucidation of novel 13‐series resolvins that increase with atorvastatin and clear infections. Nat Med 21: 1071–1075. 26236990 10.1038/nm.3911 PMC4560998

[bph14336-bib-0024] Dalli J , Colas RA , Arnardottir H , Serhan CN (2017). Vagal regulation of group 3 innate lymphoid cells and the immunoresolvent PCTR1 controls infection resolution. Immunity 46: 92–105. 28065837 10.1016/j.immuni.2016.12.009 PMC5283610

[bph14336-bib-0025] Dalli J , Colas RA , Serhan CN (2013). Novel n‐3 immunoresolvents: structures and actions. Sci Rep 3: 1940. 23736886 10.1038/srep01940 PMC3672887

[bph14336-bib-0026] Dalli J , Ramon S , Norris PC , Colas RA , Serhan CN (2015b). Novel proresolving and tissue‐regenerative resolvin and protectin sulfido‐conjugated pathways. FASEB J 29: 2120–2136. 25713027 10.1096/fj.14-268441 PMC4415017

[bph14336-bib-0027] Dalli J , Winkler JW , Colas RA , Arnardottir H , Cheng CY , Chiang N *et al* (2013a). Resolvin D3 and aspirin‐triggered resolvin D3 are potent immunoresolvents. Chem Biol 20: 188–201. 23438748 10.1016/j.chembiol.2012.11.010 PMC3583372

[bph14336-bib-0028] Dalli J , Zhu M , Vlasenko NA , Deng B , Haeggstrom JZ , Petasis NA *et al* (2013b). The novel 13S,14S‐epoxy‐maresin is converted by human macrophages to maresin 1 (MaR1), inhibits leukotriene A4 hydrolase (LTA4H), and shifts macrophage phenotype. FASEB J 27: 2573–2583. 23504711 10.1096/fj.13-227728 PMC3688739

[bph14336-bib-0029] de Oliveira JR , da Silva PR , Rogerio AP (2017). AT‐RvD1 modulates the activation of bronchial epithelial cells induced by lipopolysaccharide and Dermatophagoides pteronyssinus. Eur J Pharmacol 805: 46–50. 28322829 10.1016/j.ejphar.2017.03.029

[bph14336-bib-0030] Deng B , Wang CW , Arnardottir HH , Li Y , Cheng CY , Dalli J *et al* (2014). Maresin biosynthesis and identification of maresin 2, a new anti‐inflammatory and pro‐resolving mediator from human macrophages. PLoS One 9: e102362. 25036362 10.1371/journal.pone.0102362 PMC4103848

[bph14336-bib-0031] Deyama S , Shimoda K , Suzuki H , Ishikawa Y , Ishimura K , Fukuda H *et al* (2018). Resolvin E1/E2 ameliorate lipopolysaccharide‐induced depression‐like behaviors via ChemR23. Psychopharmacology (Berl) 235: 329–336. 29090333 10.1007/s00213-017-4774-7

[bph14336-bib-0032] Dinarello CA , Joosten LA (2016). Inflammation in rheumatology in 2015: new tools to tackle inflammatory arthritis. Nat Rev Rheumatol 12: 78–80. 26763730 10.1038/nrrheum.2015.180

[bph14336-bib-0033] El Kebir D , Gjorstrup P , Filep JG (2012). Resolvin E1 promotes phagocytosis‐induced neutrophil apoptosis and accelerates resolution of pulmonary inflammation. Proc Natl Acad Sci U S A 109: 14983–14988. 22927428 10.1073/pnas.1206641109 PMC3443132

[bph14336-bib-0034] Fiore S , Maddox JF , Perez HD , Serhan CN (1994). Identification of a human cDNA encoding a functional high affinity lipoxin A4 receptor. J Exp Med 180: 253–260. 8006586 10.1084/jem.180.1.253 PMC2191537

[bph14336-bib-0035] Fiore S , Serhan CN (1995). Lipoxin A4 receptor activation is distinct from that of the formyl peptide receptor in myeloid cells: inhibition of CD11/18 expression by lipoxin A4‐lipoxin A4 receptor interaction. Biochemistry 34: 16678–16686. 8527441 10.1021/bi00051a016

[bph14336-bib-0036] Fonseca FC , Orlando RM , Turchetti‐Maia RM , de Francischi JN (2017). Comparative effects of the omega3 polyunsaturated fatty acid derivatives resolvins E1 and D1 and protectin DX in models of inflammation and pain. J Inflamm Res 10: 119–133. 28919798 10.2147/JIR.S142424 PMC5587166

[bph14336-bib-0037] Francos‐Quijorna I , Santos‐Nogueira E , Gronert K , Sullivan AB , Kopp MA , Brommer B *et al* (2017). Maresin 1 promotes inflammatory resolution, neuroprotection, and functional neurological recovery after spinal cord injury. J Neurosci 37: 11731–11743. 29109234 10.1523/JNEUROSCI.1395-17.2017 PMC5707767

[bph14336-bib-0038] Freire MO , Dalli J , Serhan CN , Van Dyke TE (2017). Neutrophil resolvin E1 receptor expression and function in type 2 diabetes. J Immunol 198: 718–728. 27994073 10.4049/jimmunol.1601543 PMC5224973

[bph14336-bib-0039] Fukuda H , Muromoto R , Takakura Y , Ishimura K , Kanada R , Fushihara D *et al* (2016). Design and synthesis of cyclopropane congeners of resolvin E2, an endogenous proresolving lipid mediator, as its stable equivalents. Org Lett 18: 6224–6227. 27978690 10.1021/acs.orglett.6b02612

[bph14336-bib-0040] Fukunaga K , Kohli P , Bonnans C , Fredenburgh LE , Levy BD (2005). Cyclooxygenase 2 plays a pivotal role in the resolution of acute lung injury. J Immunol 174: 5033–5039. 15814734 10.4049/jimmunol.174.8.5033

[bph14336-bib-0041] Gewirtz AT , Collier‐Hyams LS , Young AN , Kucharzik T , Guilford WJ , Parkinson JF *et al* (2002). Lipoxin a4 analogs attenuate induction of intestinal epithelial proinflammatory gene expression and reduce the severity of dextran sodium sulfate‐induced colitis. J Immunol 168: 5260–5267. 11994483 10.4049/jimmunol.168.10.5260

[bph14336-bib-0042] Gilroy DW , Colville‐Nash PR , Willis D , Chivers J , Paul‐Clark MJ , Willoughby DA (1999). Inducible cyclooxygenase may have anti‐inflammatory properties. Nat Med 5: 698–701. 10371510 10.1038/9550

[bph14336-bib-0043] Gobbetti T , Dalli J , Colas RA , Federici Canova D , Aursnes M , Bonnet D *et al* (2017). Protectin D1n‐3 DPA and resolvin D5n‐3 DPA are effectors of intestinal protection. Proc Natl Acad Sci U S A 114: 3963–3968. 28356517 10.1073/pnas.1617290114 PMC5393238

[bph14336-bib-0044] Goldstein JL , Cryer B (2015). Gastrointestinal injury associated with NSAID use: a case study and review of risk factors and preventative strategies. Drug Healthc Patient Saf 7: 31–41. 25653559 10.2147/DHPS.S71976 PMC4310346

[bph14336-bib-0045] Guilford WJ , Bauman JG , Skuballa W , Bauer S , Wei GP , Davey D *et al* (2004). Novel 3‐oxa lipoxin A4 analogues with enhanced chemical and metabolic stability have anti‐inflammatory activity in vivo. J Med Chem 47: 2157–2165. 15056011 10.1021/jm030569l

[bph14336-bib-0046] Harding SD , Sharman JL , Faccenda E , Southan C , Pawson AJ , Ireland S *et al* (2018). The IUPHAR/BPS Guide to PHARMACOLOGY in 2018: updates and expansion to encompass the new guide to IMMUNOPHARMACOLOGY. Nucleic Acids Res 46: D1091–D1106. 29149325 10.1093/nar/gkx1121 PMC5753190

[bph14336-bib-0047] Hayhoe RP , Kamal AM , Solito E , Flower RJ , Cooper D , Perretti M (2006). Annexin 1 and its bioactive peptide inhibit neutrophil‐endothelium interactions under flow: indication of distinct receptor involvement. Blood 107: 2123–2130. 16278303 10.1182/blood-2005-08-3099

[bph14336-bib-0048] Herova M , Schmid M , Gemperle C , Hersberger M (2015). ChemR23, the receptor for chemerin and resolvin E1, is expressed and functional on M1 but not on M2 macrophages. J Immunol 194: 2330–2337. 25637017 10.4049/jimmunol.1402166

[bph14336-bib-0049] Hodges RR , Li D , Shatos MA , Serhan CN , Dartt DA (2016). Lipoxin A4 counter‐regulates histamine‐stimulated glycoconjugate secretion in conjunctival goblet cells. Sci Rep 6: 36124. 27824117 10.1038/srep36124 PMC5099697

[bph14336-bib-0050] Hua J , Jin Y , Chen Y , Inomata T , Lee H , Chauhan SK *et al* (2014). The resolvin D1 analogue controls maturation of dendritic cells and suppresses alloimmunity in corneal transplantation. Invest Ophthalmol Vis Sci 55: 5944–5951. 25146982 10.1167/iovs.14-14356 PMC4172301

[bph14336-bib-0051] Isobe Y , Arita M , Iwamoto R , Urabe D , Todoroki H , Masuda K *et al* (2013). Stereochemical assignment and anti‐inflammatory properties of the omega‐3 lipid mediator resolvin E3. J Biochem 153: 355–360. 23293324 10.1093/jb/mvs151

[bph14336-bib-0052] Isobe Y , Arita M , Matsueda S , Iwamoto R , Fujihara T , Nakanishi H *et al* (2012). Identification and structure determination of novel anti‐inflammatory mediator resolvin E3, 17,18‐dihydroxyeicosapentaenoic acid. J Biol Chem 287: 10525–10534. 22275352 10.1074/jbc.M112.340612 PMC3322993

[bph14336-bib-0053] Jo YY , Lee JY , Park CK (2016). Resolvin E1 inhibits substance P‐induced potentiation of TRPV1 in primary sensory neurons. Mediators Inflamm 2016: 5259321. 27738388 10.1155/2016/5259321 PMC5055963

[bph14336-bib-0054] Krishnamoorthy S , Recchiuti A , Chiang N , Fredman G , Serhan CN (2012). Resolvin D1 receptor stereoselectivity and regulation of inflammation and proresolving microRNAs. Am J Pathol 180: 2018–2027. 22449948 10.1016/j.ajpath.2012.01.028 PMC3349829

[bph14336-bib-0055] Kumar V , Abbas AK , Aster JC (2014). Robbins and Cotran Pathologic Basis of Disease, Ninth edn., Elsevier: Philadelphia USA.

[bph14336-bib-0056] Lee HJ , Park MK , Lee EJ , Lee CH (2013). Resolvin D1 inhibits TGF‐beta1‐induced epithelial mesenchymal transition of A549 lung cancer cells via lipoxin A4 receptor/formyl peptide receptor 2 and GPR32. Int J Biochem Cell Biol 45: 2801–2807. 24120851 10.1016/j.biocel.2013.09.018

[bph14336-bib-0057] Leonard MO , Hannan K , Burne MJ , Lappin DW , Doran P , Coleman P *et al* (2002). 15‐Epi‐16‐(para‐fluorophenoxy)‐lipoxin A(4)‐methyl ester, a synthetic analogue of 15‐epi‐lipoxin A(4), is protective in experimental ischemic acute renal failure. J Am Soc Nephrol 13: 1657–1662. 12039996 10.1097/01.asn.0000015795.74094.91

[bph14336-bib-0058] Levy BD , Clish CB , Schmidt B , Gronert K , Serhan CN (2001). Lipid mediator class switching during acute inflammation: signals in resolution. Nat Immunol 2: 612–619. 11429545 10.1038/89759

[bph14336-bib-0059] Levy BD , De Sanctis GT , Devchand PR , Kim E , Ackerman K , Schmidt BA *et al* (2002). Multi‐pronged inhibition of airway hyper‐responsiveness and inflammation by lipoxin A(4). Nat Med 8: 1018–1023. 12172542 10.1038/nm748

[bph14336-bib-0060] Levy BD , Kohli P , Gotlinger K , Haworth O , Hong S , Kazani S *et al* (2007). Protectin D1 is generated in asthma and dampens airway inflammation and hyperresponsiveness. J Immunol 178: 496–502. 17182589 10.4049/jimmunol.178.1.496 PMC3005704

[bph14336-bib-0061] Livne‐Bar I , Wei J , Liu HH , Alqawlaq S , Won GJ , Tuccitto A *et al* (2017). Astrocyte‐derived lipoxins A4 and B4 promote neuroprotection from acute and chronic injury. J Clin Invest 127: 4403–4414. 29106385 10.1172/JCI77398 PMC5707141

[bph14336-bib-0062] Lukiw WJ , Cui JG , Marcheselli VL , Bodker M , Botkjaer A , Gotlinger K *et al* (2005). A role for docosahexaenoic acid‐derived neuroprotectin D1 in neural cell survival and Alzheimer disease. J Clin Invest 115: 2774–2783. 16151530 10.1172/JCI25420 PMC1199531

[bph14336-bib-0063] Majno G (1991). The ancient riddle of sigma eta psi iota sigma (sepsis). J Infect Dis 163: 937–945. 2019770 10.1093/infdis/163.5.937

[bph14336-bib-0064] Malagoli D (2016). The Evolution of the Immune System: Conservation and Diversification, Elsevier: London, UK.

[bph14336-bib-0065] Markworth JF , Kaur G , Miller EG , Larsen AE , Sinclair AJ , Maddipati KR *et al* (2016). Divergent shifts in lipid mediator profile following supplementation with n‐3 docosapentaenoic acid and eicosapentaenoic acid. FASEB J 30: 3714–3725. 27461565 10.1096/fj.201600360R PMC5067251

[bph14336-bib-0066] Minozzi S , Bonovas S , Lytras T , Pecoraro V , Gonzalez‐Lorenzo M , Bastiampillai AJ *et al* (2016). Risk of infections using anti‐TNF agents in rheumatoid arthritis, psoriatic arthritis, and ankylosing spondylitis: a systematic review and meta‐analysis. Expert Opin Drug Saf 15: 11–34. 27924643 10.1080/14740338.2016.1240783

[bph14336-bib-0067] Mitchell S , Thomas G , Harvey K , Cottell D , Reville K , Berlasconi G *et al* (2002). Lipoxins, aspirin‐triggered epi‐lipoxins, lipoxin stable analogues, and the resolution of inflammation: stimulation of macrophage phagocytosis of apoptotic neutrophils in vivo. J Am Soc Nephrol 13: 2497–2507. 12239238 10.1097/01.asn.0000032417.73640.72

[bph14336-bib-0068] Miyata J , Fukunaga K , Iwamoto R , Isobe Y , Niimi K , Takamiya R *et al* (2013). Dysregulated synthesis of protectin D1 in eosinophils from patients with severe asthma. J Allergy Clin Immunol 131: 353–360 e351–e352. 23006546 10.1016/j.jaci.2012.07.048

[bph14336-bib-0069] Mottola G , Chatterjee A , Wu B , Chen M , Conte MS (2017). Aspirin‐triggered resolvin D1 attenuates PDGF‐induced vascular smooth muscle cell migration via the cyclic adenosine monophosphate/protein kinase A (cAMP/PKA) pathway. PLoS One 12: e0174936. 28362840 10.1371/journal.pone.0174936 PMC5376330

[bph14336-bib-0070] Mukherjee PK , Marcheselli VL , Serhan CN , Bazan NG (2004). Neuroprotectin D1: a docosahexaenoic acid‐derived docosatriene protects human retinal pigment epithelial cells from oxidative stress. Proc Natl Acad Sci U S A 101: 8491–8496. 15152078 10.1073/pnas.0402531101 PMC420421

[bph14336-bib-0071] Norling LV , Spite M , Yang R , Flower RJ , Perretti M , Serhan CN (2011). Cutting edge: humanized nano‐proresolving medicines mimic inflammation‐resolution and enhance wound healing. J Immunol 186: 5543–5547. 21460209 10.4049/jimmunol.1003865 PMC3145138

[bph14336-bib-0072] Oh SF , Dona M , Fredman G , Krishnamoorthy S , Irimia D , Serhan CN (2012). Resolvin E2 formation and impact in inflammation resolution. J Immunol 188: 4527–4534. 22450811 10.4049/jimmunol.1103652 PMC3331964

[bph14336-bib-0073] Orr SK , Colas RA , Dalli J , Chiang N , Serhan CN (2015). Proresolving actions of a new resolvin D1 analog mimetic qualifies as an immunoresolvent. Am J Physiol Lung Cell Mol Physiol 308: L904–L911. 25770181 10.1152/ajplung.00370.2014 PMC4421783

[bph14336-bib-0074] Ortiz‐Munoz G , Mallavia B , Bins A , Headley M , Krummel MF , Looney MR (2014). Aspirin‐triggered 15‐epi‐lipoxin A4 regulates neutrophil‐platelet aggregation and attenuates acute lung injury in mice. Blood 124: 2625–2634. 25143486 10.1182/blood-2014-03-562876 PMC4208278

[bph14336-bib-0075] Pamplona FA , Ferreira J , Menezes de Lima O Jr , Duarte FS , Bento AF , Forner S *et al* (2012). Anti‐inflammatory lipoxin A4 is an endogenous allosteric enhancer of CB1 cannabinoid receptor. Proc Natl Acad Sci U S A 109: 21134–21139. 23150578 10.1073/pnas.1202906109 PMC3529012

[bph14336-bib-0076] Primdahl KG , Aursnes M , Walker ME , Colas RA , Serhan CN , Dalli J *et al* (2016). Synthesis of 13(R)‐Hydroxy‐7Z,10Z,13R,14E,16Z,19Z docosapentaenoic acid (13R‐HDPA) and its biosynthetic conversion to the 13‐series resolvins. J Nat Prod 79: 2693–2702. 27704804 10.1021/acs.jnatprod.6b00634 PMC5149404

[bph14336-bib-0077] Pruss H , Rosche B , Sullivan AB , Brommer B , Wengert O , Gronert K *et al* (2013). Proresolution lipid mediators in multiple sclerosis – differential, disease severity‐dependent synthesis – a clinical pilot trial. PLoS One 8: e55859. 23409068 10.1371/journal.pone.0055859 PMC3568070

[bph14336-bib-0078] Rajasagi NK , Reddy PB , Mulik S , Gjorstrup P , Rouse BT (2013). Neuroprotectin D1 reduces the severity of herpes simplex virus‐induced corneal immunopathology. Invest Ophthalmol Vis Sci 54: 6269–6279. 23942967 10.1167/iovs.13-12152 PMC3776714

[bph14336-bib-0079] Rathod KS , Kapil V , Velmurugan S , Khambata RS , Siddique U , Khan S *et al* (2017). Accelerated resolution of inflammation underlies sex differences in inflammatory responses in humans. J Clin Invest 127: 169–182. 27893465 10.1172/JCI89429 PMC5199722

[bph14336-bib-0080] Rius B , Duran‐Guell M , Flores‐Costa R , Lopez‐Vicario C , Lopategi A , Alcaraz‐Quiles J *et al* (2017). The specialized proresolving lipid mediator maresin 1 protects hepatocytes from lipotoxic and hypoxia‐induced endoplasmic reticulum stress. FASEB J 31: 5384–5398. 28768719 10.1096/fj.201700394R

[bph14336-bib-0081] Robbins SL , Cotran RS (1979). Pathologic Basis of Disease, 2nd edn. W. B. Saunders Co.: Philadelphia.

[bph14336-bib-0082] Romano M , Cianci E , Simiele F , Recchiuti A (2015). Lipoxins and aspirin‐triggered lipoxins in resolution of inflammation. Eur J Pharmacol 760: 49–63. 25895638 10.1016/j.ejphar.2015.03.083

[bph14336-bib-0083] Rovira X , Pin JP , Giraldo J (2010). The asymmetric/symmetric activation of GPCR dimers as a possible mechanistic rationale for multiple signalling pathways. Trends Pharmacol Sci 31: 15–21. 19963287 10.1016/j.tips.2009.10.008

[bph14336-bib-0084] Samuelsson B (2012). Role of basic science in the development of new medicines: examples from the eicosanoid field. J Biol Chem 287: 10070–10080. 22318727 10.1074/jbc.X112.351437 PMC3323017

[bph14336-bib-0085] Schmid M , Gemperle C , Rimann N , Hersberger M (2016). Resolvin D1 polarizes primary human macrophages toward a proresolution phenotype through GPR32. J Immunol 196: 3429–3437. 26969756 10.4049/jimmunol.1501701

[bph14336-bib-0086] Schottelius AJ , Giesen C , Asadullah K , Fierro IM , Colgan SP , Bauman J *et al* (2002). An aspirin‐triggered lipoxin A4 stable analog displays a unique topical anti‐inflammatory profile. J Immunol 169: 7063–7070. 12471142 10.4049/jimmunol.169.12.7063

[bph14336-bib-0087] Schwab JM , Chiang N , Arita M , Serhan CN (2007). Resolvin E1 and protectin D1 activate inflammation‐resolution programmes. Nature 447: 869–874. 17568749 10.1038/nature05877 PMC2757086

[bph14336-bib-0088] Serhan CN , Chiang N , Dalli J (2017). New pro‐resolving n‐3 mediators bridge resolution of infectious inflammation to tissue regeneration. Mol Aspects Med . 10.1016/j.mam.2017.08.002 PMC5832503 28802833

[bph14336-bib-0089] Serhan CN , Clish CB , Brannon J , Colgan SP , Chiang N , Gronert K (2000). Novel functional sets of lipid‐derived mediators with antiinflammatory actions generated from omega‐3 fatty acids via cyclooxygenase 2‐nonsteroidal antiinflammatory drugs and transcellular processing. J Exp Med 192: 1197–1204. 11034610 10.1084/jem.192.8.1197 PMC2195872

[bph14336-bib-0090] Serhan CN , Dalli J , Karamnov S , Choi A , Park CK , Xu ZZ *et al* (2012). Macrophage proresolving mediator maresin 1 stimulates tissue regeneration and controls pain. FASEB J 26: 1755–1765. 22253477 10.1096/fj.11-201442 PMC3316905

[bph14336-bib-0091] Serhan CN , Hamberg M , Samuelsson B (1984). Trihydroxytetraenes: a novel series of compounds formed from arachidonic acid in human leukocytes. Biochem Biophys Res Commun 118: 943–949. 6422933 10.1016/0006-291x(84)91486-4

[bph14336-bib-0092] Serhan CN , Hong S , Gronert K , Colgan SP , Devchand PR , Mirick G *et al* (2002). Resolvins: a family of bioactive products of omega‐3 fatty acid transformation circuits initiated by aspirin treatment that counter proinflammation signals. J Exp Med 196: 1025–1037. 12391014 10.1084/jem.20020760 PMC2194036

[bph14336-bib-0093] Serhan CN , Yang R , Martinod K , Kasuga K , Pillai PS , Porter TF *et al* (2009). Maresins: novel macrophage mediators with potent antiinflammatory and proresolving actions. J Exp Med 206: 15–23. 19103881 10.1084/jem.20081880 PMC2626672

[bph14336-bib-0094] Takano T , Clish CB , Gronert K , Petasis N , Serhan CN (1998). Neutrophil‐mediated changes in vascular permeability are inhibited by topical application of aspirin‐triggered 15‐epi‐lipoxin A4 and novel lipoxin B4 stable analogues. J Clin Invest 101: 819–826. 9466977 10.1172/JCI1578 PMC508630

[bph14336-bib-0095] Takano T , Fiore S , Maddox JF , Brady HR , Petasis NA , Serhan CN (1997). Aspirin‐triggered 15‐epi‐lipoxin A4 (LXA4) and LXA4 stable analogues are potent inhibitors of acute inflammation: evidence for anti‐inflammatory receptors. J Exp Med 185: 1693–1704. 9151906 10.1084/jem.185.9.1693 PMC2196289

[bph14336-bib-0096] Torricelli AA , Santhanam A , Agrawal V , Wilson SE (2014). Resolvin E1 analog RX‐10045 0.1% reduces corneal stromal haze in rabbits when applied topically after PRK. Mol Vis 20: 1710–1716. 25558174 PMC4279593

[bph14336-bib-0097] Tungen JE , Aursnes M , Dalli J , Arnardottir H , Serhan CN , Hansen TV (2014). Total synthesis of the anti‐inflammatory and pro‐resolving lipid mediator MaR1n‐3 DPA utilizing an sp(3) ‐sp(3) Negishi cross‐coupling reaction. Chemistry 20: 14575–14578. 25225129 10.1002/chem.201404721 PMC4232850

[bph14336-bib-0098] Van Dyke TE , Hasturk H , Kantarci A , Freire MO , Nguyen D , Dalli J *et al* (2015). Proresolving nanomedicines activate bone regeneration in periodontitis. J Dent Res 94: 148–156. 25389003 10.1177/0022034514557331 PMC4270812

[bph14336-bib-0099] Viola JR , Lemnitzer P , Jansen Y , Csaba G , Winter C , Neideck C *et al* (2016). Resolving lipid mediators maresin 1 and resolvin D2 prevent atheroprogression in mice. Circ Res 119: 1030–1038. 27531933 10.1161/CIRCRESAHA.116.309492

[bph14336-bib-0100] Walker ME , Souza PR , Colas RA , Dalli J (2017). 13‐Series resolvins mediate the leukocyte‐platelet actions of atorvastatin and pravastatin in inflammatory arthritis. FASEB J 31: 3636–3648. 28465323 10.1096/fj.201700268 PMC5503705

[bph14336-bib-0101] Wang ZF , Li Q , Liu SB , Mi WL , Hu S , Zhao J *et al* (2014). Aspirin‐triggered Lipoxin A4 attenuates mechanical allodynia in association with inhibiting spinal JAK2/STAT3 signaling in neuropathic pain in rats. Neuroscience 273: 65–78. 24836854 10.1016/j.neuroscience.2014.04.052

[bph14336-bib-0102] Winkler JW , Orr SK , Dalli J , Cheng CY , Sanger JM , Chiang N *et al* (2016). Resolvin D4 stereoassignment and its novel actions in host protection and bacterial clearance. Sci Rep 6: 18972. 26743932 10.1038/srep18972 PMC4705531

[bph14336-bib-0103] Wire B (2009). Resolvyx announces positive data from Phase 2 clinical trial of the reesolvin RX‐10045 in patients with dry eye syndrome. Available at: http://www. businesswire.com/news/home/20090824005320/en/Resolvyx‐AnnouncesPositive‐Data‐Phase‐20090824005322‐Clinical.

[bph14336-bib-0104] Xu ZZ , Zhang L , Liu T , Park JY , Berta T , Yang R *et al* (2010). Resolvins RvE1 and RvD1 attenuate inflammatory pain via central and peripheral actions. Nat Med 16: 592–597 591p following 597. 20383154 10.1038/nm.2123 PMC2866054

[bph14336-bib-0105] Yamada T , Tani Y , Nakanishi H , Taguchi R , Arita M , Arai H (2011). Eosinophils promote resolution of acute peritonitis by producing proresolving mediators in mice. FASEB J 25: 561–568. 20959515 10.1096/fj.10-170027

[bph14336-bib-0106] Zhang MJ , Sansbury BE , Hellmann J , Baker JF , Guo L , Parmer CM *et al* (2016). Resolvin D2 enhances postischemic revascularization while resolving inflammation. Circulation 134: 666–680. 27507404 10.1161/CIRCULATIONAHA.116.021894 PMC5214591

